# Protective role of alpha-lipoic acid against rhabdomyolysis-induced acute kidney injury in rats

**DOI:** 10.22038/IJBMS.2024.74864.16252

**Published:** 2024

**Authors:** Sadaf Nouripour, Soghra Mehri, Tahereh Aminifard, Arezoo Hosseini, Abolfazl Khajavi Rad, Amirhossein Jafarian, Hossein Hosseinzadeh

**Affiliations:** 1 Department of Pharmacodynamics and Toxicology, School of Pharmacy, Mashhad University of Medical Sciences, Mashhad, Iran; 2 Pharmaceutical Research Center, Pharmaceutical Technology Institute, Mashhad University of Medical Sciences, Mashhad, Iran; 3 Department of Physiology, School of Medicine, Mashhad University of Medical Sciences, Mashhad, Iran; 4 Department of Pathology, Ghaem Hospital, Mashhad University of Medical Sciences, Mashhad, Iran

**Keywords:** Acute renal injury, Alpha-lipoic acid, Anti-inflammatory, Anti-oxidant, Rhabdomyolysis

## Abstract

**Objective(s):**

Rhabdomyolysis, a potentially life-threatening condition, occurs when myoglobin is released from damaged muscle cells, leading to acute kidney injury (AKI). Alpha lipoic acid (ALA), an organosulfur compound known for its anti-oxidant and anti-inflammatory properties, was examined in this study for its potential impact on rhabdomyolysis-induced AKI in rats.

**Materials and Methods:**

Six groups of rats were included in the study, with each group consisting of six rats (n=6): Control, rhabdomyolysis, rhabdomyolysis treated with different doses of ALA (5, 10, and 20 mg/kg), and ALA alone (20 mg/kg) groups. Rhabdomyolysis was induced by intramuscular injection of glycerol on the first day of the experiment, while ALA was administered intraperitoneally for four consecutive days. Renal function parameters, oxidative stress markers, and histological changes in the kidneys were evaluated. Western blot analysis was performed to measure the levels of neutrophil gelatinase-associated lipocalin (NGAL) and tumor necrosis factor-alpha (TNF-α) proteins.

**Results:**

A significant increase in serum urea, creatinine, renal malondialdehyde, NGAl, and TNF-α protein levels was observed in glycerol-injected rats. In addition, a significant decrease in glutathione was recorded. Compared to the rhabdomyolysis group, treatment with ALA recovered kidney histological and biochemical abnormalities.

**Conclusion:**

Results suggest that rhabdomyolysis-induced AKI is associated with increased oxidative stress and inflammation. Treatment with ALA improved kidney histological abnormalities and reduced oxidative stress markers in rats. Therefore, ALA may have a potential protective effect against rhabdomyolysis-induced AKI.

## Introduction

Rhabdomyolysis is a medical condition that presents as the breakdown of skeletal muscle tissue upon direct injury, resulting in the release of intracellular compounds into the bloodstream and subsequent development of systemic complications (1). The incidence of acute kidney injury (AKI) associated with rhabdomyolysis ranges from 10% to 55% among patients, making it the most serious consequence of this syndrome (2, 3). Rhabdomyolysis-associated AKI induces renal structural alterations, including glomerulosclerosis and fibrosis, and is frequently related to poor outcomes (3). In a murine experimental model, rhabdomyolysis-induced AKI can be caused by the administration of a single intramuscular injection of glycerol (4-6). The primary cause of renal injury in rhabdomyolysis is myoglobinuria, which is also a hallmark of glycerol-induced AKI. In addition to myoglobinuria, several other factors play a role in the progression of glycerol-induced renal failure. These include increased renal vasoconstriction, ischemic injury to the tubules, tubular obstruction, and the direct toxic effects of heme proteins. (6-8). Despite this, the mechanisms by which rhabdomyolysis impairs renal function are not yet fully discovered (9). Available experimental evidence indicates that the cellular release of myoglobin leads to the uncontrolled generation of reactive oxygen species (ROS), which in turn triggers inflammation events and mitochondrial disruption (5, 10, 11). Furthermore, myoglobin itself may induce lipid peroxidation and increase the production of isoprostanes (9). Previous experimental studies have provided evidence that natural anti-oxidants can recover or prevent AKI-associated tissue injury. This is hypothesized to occur following excessive free radical scavenging and lipid peroxidation inhibition (12, 13). From a chemical perspective, Alpha lipoic acid (ALA) (1,2-dithiolane-3-pentanoic acid) is a compound derived from octanoic acid that possesses a disulfide bond within its oxidized state. The presence of two sulfur atoms within the 1,2-dithiolane ring grants ALA a strong inclination towards reducing other molecules sensitive to redox reactions (14). ALA serves as a co-factor for the mitochondrial respiratory enzyme’s pyruvate dehydrogenase and α-ketoglutarate dehydrogenase (15). Moreover, different studies indicate the efficacy of ALA in mitigating pathological conditions associated with oxidative stress. These conditions encompass a range of health issues, including cardiovascular disease, obesity, hypertension, diabetes, neurological disorders, cognitive dysfunction, glaucoma, and osteoporosis. They are often accompanied by complications such as retinopathy, neuropathy, cardiovascular diabetic autonomic neuropathy, and nephropathy (16, 17). Studies have demonstrated the anti-oxidant properties of ALA in rats afflicted with sepsis, where it effectively reduced oxidative stress in various organs including the kidney, liver, and heart (18). Furthermore, these studies have shown that ALA, a naturally occurring cellular anti-oxidant, acts as a scavenger of reactive oxygen species (ROS) and reactive nitrogen species to provide renoprotection. Due to potent anti-oxidant properties of ALA, in the present study, we investigated whether ALA has nephroprotective effects in rats with AKI induced by rhabdomyolysis.

## Materials and Methods

***Chemicals and reagents***

The chemicals utilized in this study were procured from various sources. Phosphoric acid, potassium chloride, and thiobarbituric acid were obtained from the Merck Company, Germany. Trichloroacetic acid and Alpha-lipoic acid were sourced from Tinab Shimi, Iran, while the polyvinylidene fluoride membrane was acquired from Bio-Rad, USA. Additionally, 5,5′-Dithiobis 2-nitrobenzoic acid was prepared by the Sigma Company in the USA.

***Experimental design***

Thirty-six adult male Wistar rats weighing between 200–250 g were sourced from the animal house at the School of Pharmacy, Mashhad University of Medical Sciences, Iran. The rats were housed under standard conditions, with a 12-hour light-dark cycle, temperature maintained between 21 and 24 °C, and humidity levels ranging from 40 to 60%. Ethical approval for the study was obtained from Mashhad University of Medical Sciences ethical committee (ethical number: IR.MUMS.PHARMACY.REC.1400.104), adhering to their rules and procedures regarding the use of animals in research.

Following a 2-week acclimation period, the rats were randomly divided into six groups (n=6). Before glycerol injection, the rats had unrestricted access to food but were deprived of drinking water for 16 hr (19). The control group (Group 1) received an intramuscular injection of a saline solution volume equivalent to 50% glycerol in each hind leg. Group 2 was injected intramuscularly with 50% glycerol (10 ml/kg) in each hind leg, serving as the model group. The remaining groups received the same glycerol injection as Group 2 but also received intraperitoneal injections of ALA at doses of 5, 10, and 20 mg/kg for 4 consecutive days (20). The selection of ALA doses was based on previous studies demonstrating its protective effects against tissue damage (21). The study design is summarized in **Table 1**. 

***Sample collection***

All animals were euthanized by decapitation 24 hr after the final treatment, following the injection of ketamine/xylazine. Blood serum and kidney tissues were collected for pathological and biochemical analysis (22). The right kidneys were promptly frozen in liquid nitrogen and stored at -80 °C until further analysis, while the left kidneys were fixed in 10% formalin for pathological evaluation 

***Assessing kidney function***

To assess kidney function, blood serum samples were analyzed in a laboratory to measure the levels of serum creatinine and blood urea nitrogen (BUN).

***Histopathological examination***

Paraffin blocks containing kidney tissues were subjected to hematoxylin and eosin (H&E) staining. The blocks were sectioned at 2 µm intervals. Stained samples were analyzed using an image analyzer equipped with a microscope (Olympus BX-51, Tokyo, Japan) (23). Renal damage was semi-quantitatively assessed based on criteria including the percentage of damaged renal area, tubular necrosis, protein cast formation, and glomerular atrophy. Scoring was as follows: 1. Normal, 2. Mild damage (5-25% of tubules affected), 3. Moderate damage (26–50% of tubules affected), 4. Severe damage (50–75% of tubules affected), and 5. Extensive damage (over 75% of tubules affected).

***Lipid peroxidation assay***

Malondialdehyde (MDA) levels serve as a valuable marker for assessing lipid peroxidation. Elevated MDA levels indicate an upsurge in lipid peroxidation. Under acidic conditions, malondialdehyde reacts with thiobarbituric acid to form a pink-colored complex with peak absorption at 532 nm (24). Initially, a 10% homogenate was prepared in 1.15% cold KCl. Subsequently, 0.5 ml of this homogenate was mixed with 1 ml of a 0.6% TBA solution and 3 ml of 1% phosphoric acid. The mixture was then boiled for 45 min, cooled, vortexed with 4 ml of n-butanol for one minute, and centrifuged for 10 min at 3000 rpm. The organic phase of the supernatant was transferred to new tubes, and the absorbance of each sample was measured at 532 nm. Finally, the MDA concentration was expressed as nmol/g tissue (25). 

***Determination of GSH content***

The assay utilizes the interaction between unbound sulfhydryl groups and the DTNB reagent in an alkaline condition. This interaction leads to the creation of a colored complex that has maximum absorbance at 412 nm (26). To carry out this test, tissue samples were homogenized with a phosphate buffer at pH 7.4 to create a 10% homogenate. The resulting mixture was combined with 10% TCA in a 1:1 ratio and then centrifuged at 2500 g for 10 min. The supernatant was collected and mixed with 2 ml of phosphate buffer at pH 8. Subsequently, 0.5 ml of 0.04% DTNB reagent was added to each sample, and the absorbance was measured at a wavelength of 412 nm. The amount of GSH content in each sample was then quantified and reported as nmol/g tissue (27).

***Western blot assays***

To begin the experiment, the tissue samples were homogenized in a lysis buffer. The lysis buffer consisted of the following components: 10 mM sodium azide, 1 mM sodium orthovanadate (Na_3_VO_4_), 1 mM phenylmethylsulfonyl fluoride, 10 mM glycerophosphate, 50 mM Tris-HCl (pH 7.4), 2 mM egtazic acid (EGTA), 2 mM ethylenediaminetetraacetic acid (EDTA), 0.2% W/V sodium deoxycholate, and a complete protease. The homogenate was subjected to sonication on ice, using three 10-second bursts at high intensity, with a 10-second cooling period between each burst. Afterward, it was centrifuged at 10,000 g for 10 min at 4 °C. To determine the protein content, the Bradford assay kit from BioRad (USA) was utilized. Each sample was mixed in a 1:1 volume-to-volume ratio with 2× SDS blue buffer, then boiled, aliquoted, and stored in a freezer at -80 °C (28). Samples were loaded onto a 12% gel and subjected to electrophoresis. Subsequently, they were transferred onto a PVDF membrane (BioRad, USA). The membranes were blocked for 2 hr at 37 °C using 5% nonfat milk powder (skimmed milk). Afterward, the membranes were rinsed three times with tris-buffered saline and tween 20 (TBST). Next, they were incubated overnight (16–18 hr) at 4 °C on a rocker with rabbit polyclonal anti-serum against TNF-α antibody (Cell signaling, #3707, 1: 1,000), rabbit monoclonal anti-serum against NGAL (Abcam#216462, 1: 1,000) and mouse polyclonal anti-β-actin antibodies (Cell signaling #3700S, 1: 1,000). Following three washes with TBST, the membranes were incubated with rabbit or mouse horseradish peroxidase-conjugated anti-IgG (Cell Signaling #7074, 1:3000; Cell Signaling #7076, 1:3000, respectively) for 2 hr at room temperature. The peroxidase-coated bands were visualized using enhanced chemiluminescence. The integrated optical densities of the bands were quantified using the Alliance 4.7 Gel Doc program (UK), and densitometric analysis of the protein bands was performed using the UV Tec Software package (UK). The protein concentrations were normalized relative to the corresponding bands of the control protein β-actin.

***Statistical analysis***

Pathological markers were presented as median (interquartile range, IQR), and nonparametric Kruskal-Wallis test and Dunn᾿s post-test were employed for comparisons. Other data were presented as mean ± SD. To compare the data between different groups, a one-way ANOVA test with Tukey-Kramer post-test was used. Statistical analysis was performed using GraphPad Prism 8.0 (Software Inc., San Diego, CA, USA). A *P*-value greater than 0.05 was considered statistically significant.

## Results

***Effects of ALA on glycerol-induced kidney dysfunction***

**Figure 1:**
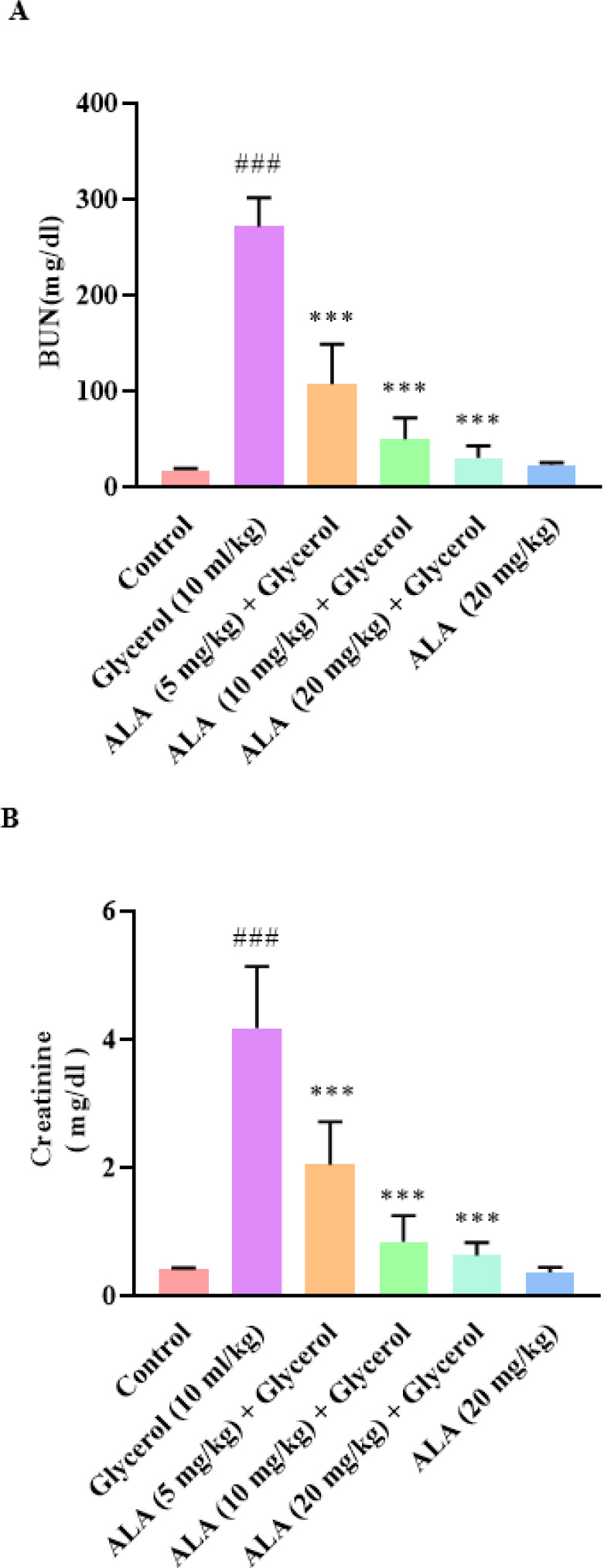
Effect of glycerol 50% (10 ml/kg) and ALA (5, 10, and 20 mg/kg) on rat serum A: BUN levels and B: Creatinine

The results indicated that glycerol injection (10 ml/kg (elevated creatinine and BUN levels compared to the control group (*P*<0.001). However, ALA treatment at doses of 5, 10, and 20 mg/kg, IP, for 4 days significantly reduced the creatinine and BUN levels (*P*<0.001 vs glycerol group). Moreover, the values of serum BUN and creatinine did not change significantly in the ALA group (20 mg/kg) compared to the control group (Figures 1A and 1B).

***Effects of ALA on glycerol-induced renal histopathology***

**Figure 2:**
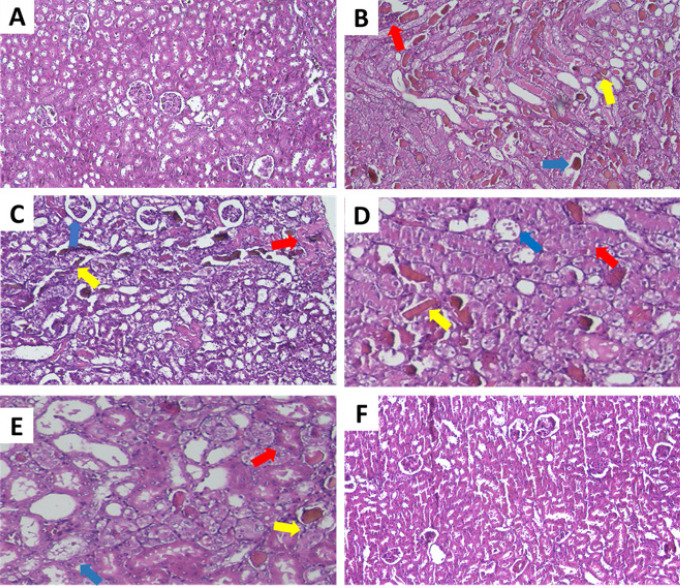
Effect of glycerol 50% (10 ml/kg) and ALA (5, 10, and 20 mg/kg) on rat kidney tissue

**Figure 3:**
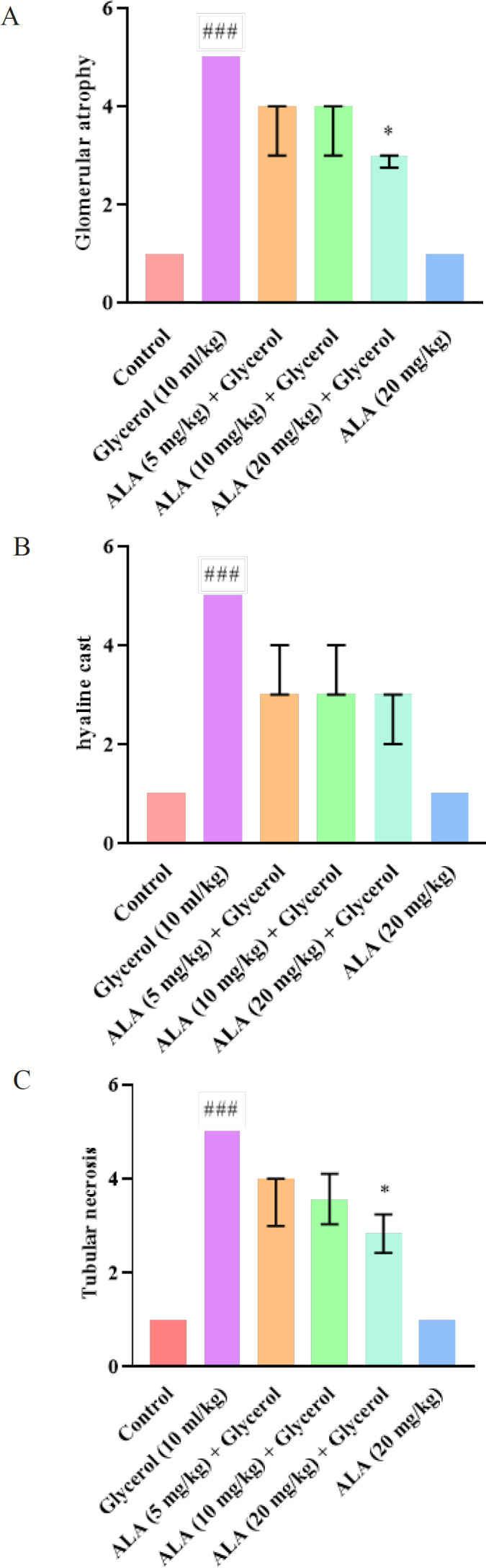
Effect of glycerol 50% (10 ml/kg) and ALA (5, 10, and 20 mg/kg) on different tissue indices in rat

According to Figures 2 and 3, the control group exhibited normal kidney tissue structure. However, glycerol injection at a dose of 10 ml/kg resulted in kidney tissue injury, specifically tubular necrosis, protein cast formation, and glomerular atrophy, compared to the control group (*P*<0.001). Treatment with ALA (20 mg/kg) intraperitoneally for 4 days alongside glycerol reduced tubular necrosis and glomerular atrophy compared to the glycerol-only group (*P*<0.05), but had no significant effect on protein cast formation. Interestingly, administration of ALA at doses of 5 and 10 mg/kg intraperitoneally for 4 days did not significantly alleviate the severity of tubular injury and renal lesions.

Additionally, histopathological analysis showed no significant difference in the ALA alone group (20 mg/kg) compared to control animals.

***Effect of ALA on glycerol-induced lipid peroxidation in kidney tissues***

**Figure 4:**
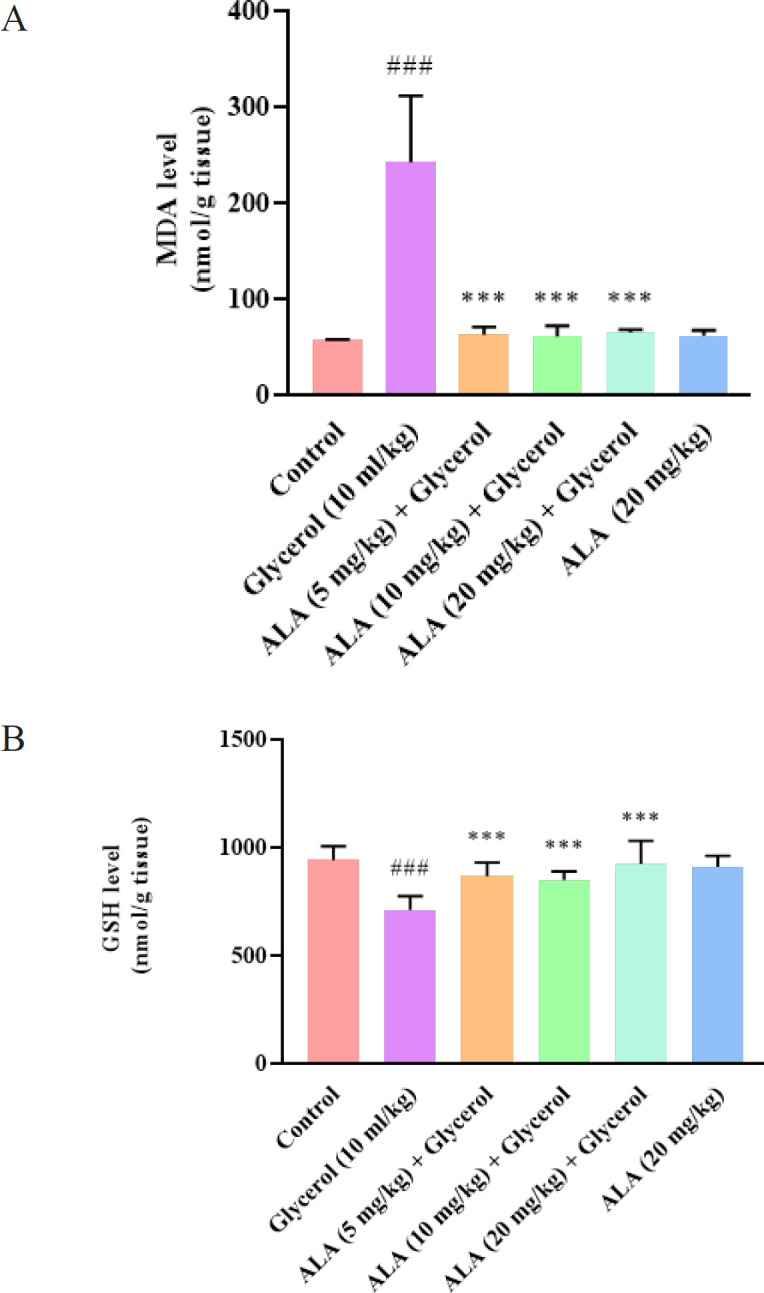
Effect of glycerol 50% (10 ml/kg) and ALA (5, 10, and 20 mg/kg) on A: MDA and B: GSH levels in the rat kidney tissue

The results demonstrated that severe lipid peroxidation in kidney tissue was confirmed by a great elevation in the MDA levels in rats receiving glycerol (10 ml/kg) compared to the control group (*P*<0.001). Conversely, after co-administration of ALA at doses of 5, 10, and 20 mg/kg with glycerol, the MDA levels in kidney tissue were meaningfully reduced compared to the glycerol group (*P*<0.001). Moreover, ALA administration alone at a dose of 20 mg/kg did not cause a remarkable change in the MDA level compared to the control group (Figure 4A).

***Effect of ALA on glycerol-induced glutathione reduction in kidney tissues***

As shown in Figure 4B, our findings demonstrate that the kidney GSH content was considerably lower in the glycerol-administered group compared to control rats (*P*<0.001). Co-administration of ALA at doses of 5, 10, and 20 mg/kg with glycerol meaningfully increased kidney GSH content compared to the glycerol group (*P*<0.001). Notably, ALA (20 mg/kg) administration did not alter GSH levels when compared with control rats.

***Effect of glycerol and ALA on TNF-α and NGAL levels in kidney tissues***

**Figure 5:**
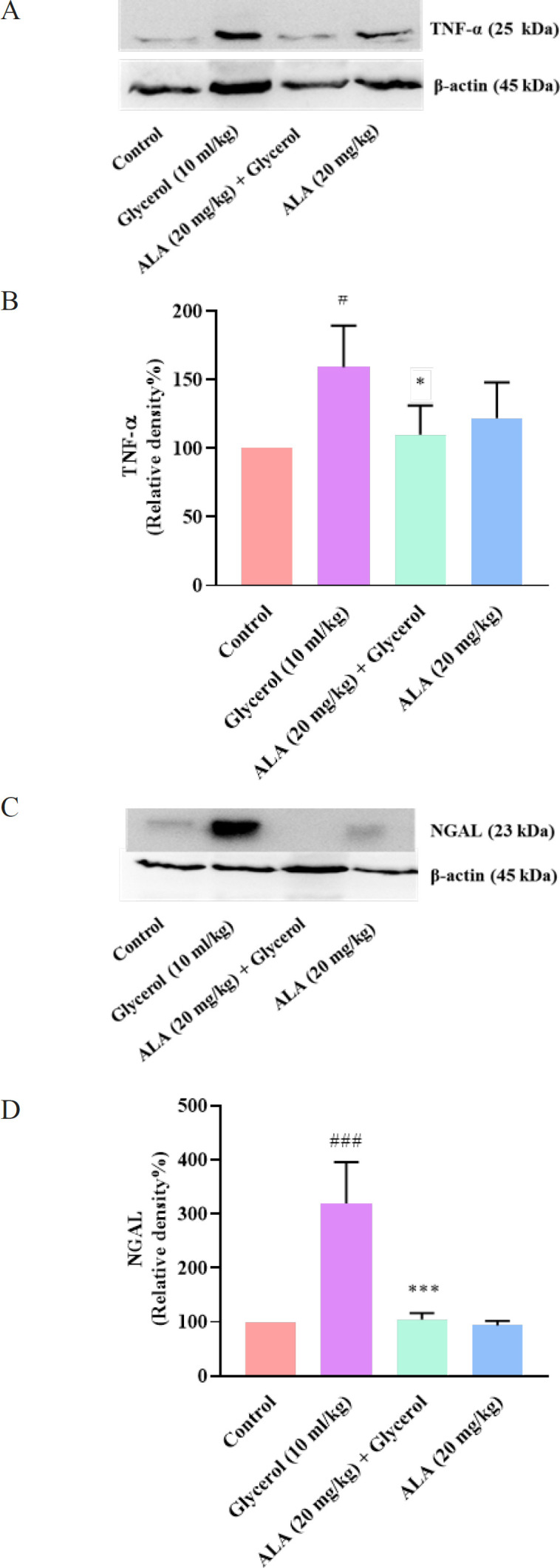
Effect of glycerol 50% (10 ml/kg) and ALA (20 mg/kg) on TNF-α and NGAL levels rat in kidney tissues. A, C: Show immunoblot bands of the western blotting analysis and B, D: Display quantitative presentation of the immunoblots

According to the results, the administration of a single dose of 10 ml/kg glycerol on the first day markedly raised the level of TNF-α protein in the rat kidney tissue compared to the control group (*P*<0.05). It is important to note that co-administering the highest dose of ALA (20 mg/kg) for 4 days with glycerol caused a significant reduction in the level of TNF-α protein in comparison to the glycerol group (*P*<0.05). However, administration of ALA (20 mg/kg) alone did not change the TNF-α level in comparison to the control rats (Figures 5A and 5B).

As presented in Figures 5C and 5D, the expression of NGAL protein in the kidney tissue was significantly increased following administration of glycerol in comparison to the control group (*P*<0.001). However, when ALA at a dose of 20 mg/kg was co-administered with glycerol, the level of NGAL meaningfully declined in the kidney tissue compared to the rats that received glycerol (*P*<0.001).

Administration of ALA (20 mg/kg) alone did not affect the level of NGAL compared to the control group. 

Data are expressed as mean±SD, (n=6). Statistical difference was examined using the ANOVA test and a Tukey-Kramer posttest. ### *P*<0.001 vs control group, *** *P*<0.001 vs glycerol group

ALA: Alpha lipoic acid.

Tissue sections were stained with hematoxylin and eosin (H&E) and observed under a light microscope magnification of 400 x (5 µm). Group A: control, B: glycerol, C: glycerol + ALA (5 mg/kg), D: glycerol + ALA (10 mg/kg), E: glycerol + ALA (20 mg/kg), and F: ALA (20 mg/kg). Red arrows indicate tubular necrosis, yellow arrows indicate hyaline cast formation, and blue arrows indicate glomerular atrophy.

ALA: Alpha lipoic acid

A: glomerular atrophy, B: hyaline cast, and C: tubular necrosis. The data are shown as non-parametric (n=6). To assess the statistical difference, the ANOVA test and non-parametric Kruskal-Wallis test were employed. ### *P*<0.001 vs control group, * *P*<0.05 vs glycerol group

ALA: Alpha lipoic acid

Data are expressed as mean±SD (n=6). Statistical difference was examined using the ANOVA test and a Tukey-Kramer posttest. ### *P*<0.001 vs control group, *** *P*<0.001 vs glycerol group

ALA: Alpha lipoic acid

Data were analyzed by one-way ANOVA following Tukey’s for multiple comparisons. # *P*<0.05 and ### *P*<0.001 vs control group, * *P*<0.05 and *** *P*<0.001 vs glycerol group

ALA: Alpha lipoic acid

## Discussion

The purpose of this research was to explore the possible nephroprotective effects of ALA on rhabdomyolysis-induced AKI in rats. According to our study findings, the injection of glycerol 50% (10 ml/kg) led to renal tubular necrosis, glomerular atrophy, and hyaline cast in the kidney. Moreover, a marked increase in serum creatinine and BUN, and kidney tissue levels of MDA, TNF-α, and NGAL was observed in our experiments. Whereas, there was a significant diminish in GSH level in the kidney. Nevertheless, co-administration of ALA at doses of 5, 10, and 20 mg/kg with glycerol 50% (10 ml/kg) declined serum creatinine, BUN, and MDA levels and increased GSH levels in the kidney. The simultaneous administration of glycerol and ALA (20 mg/kg) had a notable effect on TNF-α level and histological lesions and also reduced the level of NGAL. 

Glycerol injection is a commonly used approach to create an AKI model in experimental studies (4). In this condition, skeletal muscle degeneration leads to the release of myocyte contents into the bloodstream, eventually leading to a myoglobinuric condition that resembles clinical rhabdomyolysis (29). Previous studies have exhibited that a significant elevation in urea and creatinine levels as important markers of kidney damage was found in rats receiving glycerol which indicates the presence of AKI (30). We also found that glycerol 50% (10 ml/kg) raised serum creatinine and BUN levels after a single injection. However, it was noteworthy that co-administration of glycerol and ALA (5, 10, and 20 mg/kg, IP, for 4 days) inverted these alterations. Renoprotective effects of ALA have been reported previously which are in line with our results. ALA treatment (200 mg/kg, orally) has been shown to decrease serum creatinine and urea in septic rats (31). ALA (100 mg/kg, IP, for 5 days) ameliorated amikacin-induced renal injury in rats by reducing serum creatinine and urea levels. (32). Morphological information indicated that structural injury such as tubular necrosis, glomerular atrophy, and hyaline cast was induced in kidney tissue following glycerol injection which confirmed the previous findings (33). It has been reported that injecting glycerol 50% (10 ml/kg) caused tubular lesions such as tubule vacuolation and glomerular injury, leading to their subsequent necrosis (34).

Administration of ALA (20 mg/kg) significantly improved histological tubular injury in previous studies. According to recent research, ALA (50 mg/kg, IP, for 2 days) attenuated LPS-induced kidney injury, such as renal tubular dysfunction (35). Additionally, ALA (100 mg/kg, alone, IP) 24 hr before acute ischemic renal failure reduced all changes in renal function, including tubular necrosis, protein casts, and hemorrhage (36).

The progression of rhabdomyolysis-induced AKI is believed to be associated with the generation of oxygen-free radicals that originate from myoglobin. Several studies have provided evidence in support of this hypothesis by indicating that oxidative stress has a crucial role in rhabdomyolysis-induced AKI (37, 38). These studies have shown an increase in markers such as MDA, which indicate lipid peroxidation, and a reduction in the level of the anti-oxidant enzyme SOD in renal tissue after glycerol injection. In previous studies, we found that 50% glycerol (10 ml/kg) injection in rat muscle altered kidney tissue oxidant and anti-oxidant markers such as MDA and GSH. However, ALA at the doses of 5, 10, and 20 mg/kg was effectively able to diminish the level of MDA and elevated GSH content. Our data are in line with a recent report, which showed that ALA (100 mg/kg, IP, for 2 weeks) ameliorated cisplatin-induced proximal tubule injury of AKI by reducing structural damage, improving glomerular filtration, and reducing excretion of urinary biomarkers (39). Additionally, ALA (50 mg/kg, orally for 14 days) was able to decrease renal contents of MDA, nitric oxide, cyclooxygenase-2, and caspase-3, indicating the protective effects of ALA against doxorubicin nephrotoxicity (40).

Inflammation is the primary and major mechanism of AKI. Interestingly, we observed a rise in TNF-α expression following injection of glycerol. Various studies have demonstrated an increase in the production of pro-inflammatory cytokines in response to renal injury (41). The results of our study displayed that 4-day treatment with ALA (20 mg/kg) significantly decreased TNF-α levels in the kidney. Previously it has been shown that ALA (60 mg/kg, IP) decreased pro-inflammatory cytokine levels by inhibiting NF-kB and suppressing gene expression in lipopolysaccharide-induced oxidative stress in rat kidneys (42). 

Under usual conditions, lower NGAL expression levels can be discovered in the kidney (43). It has been considered that elevated levels of NGAL can be induced by AKI (44), so, NGAL can be mentioned as the biochemical marker for AKI diagnosis. In agreement with previous studies, we observed a substantial increase in NGAL levels in kidney tissue after intramuscular injection of 10 ml/kg glycerol 50%. Nevertheless, we also observed that administration of ALA (20 mg/kg) led to a significant decrease in NGAL levels compared to the group that received glycerol alone. In line with our results, the oral administration of ALA (200 mg/kg, for 1 day) was shown to decrease NGAL levels, suggesting the protective effects of ALA against sepsis. Sepsis, a leading cause of mortality worldwide, is often linked with multiple organ dysfunction (45).

## Conclusion

ALA recovers kidney function in rhabdomyolysis-induced AKI. ALA inhibits the oxidative challenge induced by glycerol. Additionally, ALA reduces the level of TNF-α in the renal tissue. Therefore, it can be concluded that ALA protects against the progression of AKI via its anti-oxidant and anti-inflammatory properties. 

## Authors’ Contributions

S N performed experiments, analyzed the data, and wrote the manuscript. S M supervised the study and revised the manuscript. T A analyzed the data, interpreted the results, and revised the manuscript. A H performed experiments. A KH supervised the study. A J performed the pathology tests. H H designed the study, supervised the research, and revised the manuscript. All authors approved the current version of the manuscript. 

## Conflicts of Interest

 The authors declare that they have no conflicts of interest.
